# Epidemiology of herpes zoster and postherpetic neuralgia in Japan: analysis of a large-scale claims database

**DOI:** 10.1186/s12879-026-12534-0

**Published:** 2026-01-10

**Authors:** Maho Adachi-Katayama, Naoki Kanda, Seiya Sasakura, Shuji Hatakeyama

**Affiliations:** 1 Division of Infectious Diseases, Department of Infection and Immunity, Jichi Medical University, 3311-1 Yakushiji, Shimotsuke-shi, Tochigi 329-0498 Japan; 2 Department of Infectious Diseases, The University of Tokyo Hospital, 7-3-1 Hongo, Bunkyo-ku, Tokyo, 113-8655 Japan; 3 Division of General Medicine, Jichi Medical University, 3311-1 Yakushiji, Shimotsuke-shi, Tochigi 329-0498 Japan

**Keywords:** Claims database, Herpes zoster, Postherpetic neuralgia, Varicella-zoster virus, Epidemiology

## Abstract

**Background:**

Herpes zoster (HZ) is a global public health concern. Although HZ vaccination is expected to reduce both HZ and its complications, comprehensive epidemiological data to guide vaccination strategies are scarce.

**Methods:**

We conducted a large-scale analysis of a claims database in Japan to examine standardized incidence rates of HZ and postherpetic neuralgia (PHN) from April 2014 to March 2023. We also evaluated the risks of hospitalization and PHN after HZ, and analyzed varicella incidence using national surveillance data.

**Results:**

The overall standardized incidence rates were 9.58 and 1.00 per 1,000 person-years for HZ and PHN, respectively. From 2014 to 2023, these rates increased, with estimated annual percent changes of 1.16% (95% confidence interval, 0.52–2.00) for HZ and 0.99% (0.09–3.04) for PHN. The incidence of HZ increased steadily with age: 7.17 in the 40–49 age group, 9.73 in the 50–59 age group, 13.96 in the 60–69 age group, 17.99 in the 70–79 age group, and 18.81 in those aged ≥ 80 years. The effect of aging was more pronounced for PHN, with corresponding rates of 0.37, 0.78, 1.78, 2.97, and 2.98, respectively. The overall risks of hospitalization and PHN following HZ were 4.8% and 10.4%, respectively, with risk ratios approximately 2–3 times higher in those aged ≥ 70 years than in those aged 50–59. HZ incidence was higher in summer and showed little association with varicella incidence.

**Conclusions:**

Our findings provide evidence to inform individuals and policymakers in developing strategies to prevent HZ and its complications.

**Supplementary Information:**

The online version contains supplementary material available at 10.1186/s12879-026-12534-0.

## Background

Herpes zoster (HZ) is a neurocutaneous disease caused by reactivation of the latent varicella-zoster virus (VZV) in the dorsal sensory or cranial nerve ganglia [1]. Postherpetic neuralgia (PHN) is a common complication of HZ, characterized by chronic pain, reduced quality of life, and increased healthcare costs [2]. Recent studies have also suggested associations between HZ, dementia, and cardiovascular diseases [3, 4]. In the general population, 10%–30% of individuals develop HZ during their lifetime, with risk increasing with advancing age and conditions of impaired cellular immunity, including immunocompromising diseases and immunosuppressive therapy [1, 5]. HZ affects millions of people worldwide each year, and together with its complications, represents a substantial public health concern.

Vaccination against HZ has been a major advancement in healthcare, reducing the risks of both HZ and PHN in high-risk populations [6]. The recombinant zoster vaccine, approved in October 2017 in the United States, demonstrated robust real-world effectiveness of 76% against HZ [7]. In Japan, the live-attenuated zoster vaccine was approved in March 2016, and the recombinant zoster vaccine was introduced in January 2020 for adults aged ≥ 50 years, with its indication expanded in June 2023 to include adults aged ≥ 18 years at increased risk of HZ. However, throughout the study period, HZ vaccination was not included in the national immunization program, and no nationwide public funding was available. Consequently, vaccine uptake in Japan has remained limited. As of 2022, municipal subsidies covering part of the vaccination cost were provided in only 3.2% (56/1,741) of municipalities, covering 3.7% of the population aged ≥ 50 years in Japan. Even among these subsidized populations, the reported vaccination coverage was only 3.0% [8]. To address this, the Japanese government began providing subsidies in April 2025, covering individuals aged ≥ 65 years and older and those aged ≥ 60 years and older with human immunodeficiency virus (HIV)-related severe disabilities.

Although the incidence of HZ and PHN has been reported in Japan and several other countries [9–12], large epidemiological studies comprehensively covering all age groups remain limited. This study aimed to use a large-scale, nationally representative claims database to analyze the epidemiology of HZ and PHN in Japan and to evaluate the risks of hospitalization and PHN following HZ, thereby providing evidence to guide vaccination decisions and inform healthcare policymakers in developing strategies for the prevention of HZ and its complications, including the determination of optimal vaccination age thresholds.

## Methods

### Data sources

In Japan, all citizens are covered by a universal health insurance program consisting of a social insurance system (for employees aged < 75 years), a national health insurance system (for self-employed or unemployed individuals aged < 75 years), and an advanced elderly medical service system (for those aged ≥ 75 years). This study used a large commercial claims database provided by DeSC Healthcare (Tokyo, Japan), which included data on approximately 12.5 million individuals [13]. As the database contains claims from all three types of insurers, it covers individuals across the entire age spectrum. Diagnoses were recorded according to the International Classification of Diseases and Related Health Problems, 10th Revision (ICD-10).

Claims data for individuals diagnosed with HZ (ICD-10 code B02) between April 2014 and March 2023 were extracted and analyzed. The dataset comprised monthly medical and pharmaceutical claims, including both inpatient and outpatient records. It contained information on patient demographics (year and month of birth and sex), unique individual identifiers, diagnoses, dates of diagnosis, medical procedures, and prescribed medications.

### Study design and participant selection

The incidence of HZ was defined as a diagnosis of HZ (ICD-10 code B02, excluding codes specific to PHN) accompanied by a prescription for systemic antivirals (acyclovir, amenamevir, famciclovir, valacyclovir, or vidarabine) within 3 days of diagnosis. Recurrent HZ diagnoses within 180 days were considered the same event and excluded. The incidence of PHN was defined, according to a previous study [14], as meeting either of the following criteria: (1) a diagnostic code for PHN (B022) recorded 30–180 days after the initial HZ diagnosis, or (2) a prescription for PHN-related medications issued 30–180 days after the diagnosis of HZ. PHN-related medications included opioids (tramadol), anticonvulsants (carbamazepine, gabapentin, mirogabalin, phenytoin, and pregabalin), a non-opioid/non-cyclooxygenase inhibitor analgesic (Neurotropin^®^, an extract from inflamed cutaneous tissue of rabbits inoculated with vaccinia virus), and antidepressants (amitriptyline, duloxetine, imipramine, and nortriptyline) [15–17]. Because nonsteroidal anti-inflammatory drugs (NSAIDs) and acetaminophen lack specificity for identifying PHN, they were excluded from the list of PHN-related medications [16]. Complicated HZ was defined as disseminated zoster (ICD-10 code B027); zoster ocular disease (B023); neurological complications, including zoster encephalitis, zoster meningitis, and zoster with other nervous system involvement (B020 and B021); or Ramsay–Hunt syndrome (B022). Hospitalizations occurring within 30 days of HZ diagnosis were also extracted.

Comorbidities were identified using ICD-10 codes, based on previous studies [11, 18, 19], during the period from 3 months before HZ diagnosis to the index date. These comorbidities included asthma, cardiovascular disease, chronic kidney disease, cirrhosis or hepatitis, chronic obstructive pulmonary disease, depression, diabetes mellitus, hematologic malignancies, hematopoietic stem cell transplantation, HIV infection, inflammatory bowel disease, musculoskeletal disorders, neurological disorders, other autoimmune diseases, primary immunodeficiency, psoriasis, rheumatoid arthritis, systemic lupus erythematosus, solid organ transplantation, and solid tumors. Corresponding ICD-10 codes are listed in Supplementary Table 1. Immunosuppressive medications were classified as corticosteroids, immunomodulatory agents, and biological agents according to a previous database study by Sun et al. [20]. Definitions of immunomodulatory and biological agents according to the Anatomical Therapeutic Chemical (ATC) Classification System are provided in Supplementary Table 2.

### Description of the epidemiology of HZ and complications

We analyzed the monthly and annual incidence rates of HZ and PHN, as well as hospitalizations following HZ. Age- and sex-specific incidence rates, incidence rate ratios (IRRs) for HZ and PHN, risks (proportions), and risk ratios for PHN and hospitalization after HZ were calculated. Trends in antiviral prescriptions were also examined.

The monthly incidence of varicella was evaluated separately using national sentinel surveillance data from the Infectious Diseases Surveillance Center in Japan [21]. In Japan, varicella is monitored through a national sentinel surveillance system, in which approximately 3,000 sentinel sites, including pediatric clinics and hospitals, report weekly case counts. In contrast, HZ is not subject to national surveillance, and no substantial changes in reporting or claims systems occurred during the study period.

### Statistical analysis

Crude incidence rates of HZ, PHN, and hospitalization were calculated from monthly case counts stratified by sex and age group and then converted to annual crude incidence rates per 1,000 person-years for each stratum. Age- and sex-standardized incidence rates were calculated based on the age and sex distributions of the Japanese population in 2015. Annual percent changes in the incidence rates of HZ and PHN were estimated using generalized linear regression with a Poisson distribution, and 95% confidence intervals (CIs) were derived using the bootstrap method. The IRRs and risk ratios were calculated using the 50–59-year age group as the reference. For sensitivity analyses, the incidence of HZ was alternatively defined based on diagnostic codes alone (without antiviral prescriptions), and the incidence rates were recalculated.

All statistical analyses were conducted on a fiscal year (FY) basis using two-tailed tests with a significance level of 5%. All analyses were performed using the R software (version 4.4.3; R Foundation for Statistical Computing, Vienna, Austria).

### Ethics statement

This study was conducted in accordance with the principles of the Declaration of Helsinki and approved by the Ethics Committee of Jichi Medical University (approval number: 24–112). The requirement for informed consent was waived due to the retrospective study design and the use of anonymized data.

## Results

The database included 9,063,112 insured individuals and their dependents as of 2022. Of these, 544,239 (6.0%), 2,107,716 (23.3%), and 6,411,157 (70.7%) were aged < 20 years, 20–59 years, and ≥ 60 years, respectively. The proportion of older individuals was higher than in the reference population of Japanese individuals in 2015 (Supplementary Table 3).

Between April 2014 and March 2023, we identified 727,117 episodes of HZ in 692,502 patients. Of these, 96,450 (13.9%) patients developed 99,153 PHN episodes. The demographic characteristics are shown in Table 1. The median age was 74 years among individuals with HZ and 76 years among those with PHN; 62.2% and 60.6%, respectively, were female. Musculoskeletal disorders, diabetes mellitus, and cardiovascular diseases were the most common comorbidities, and 12% of patients received corticosteroids. Among the 727,117 HZ episodes, 45,771 (6.3%) patients were hospitalized. The median interval between diagnosis and hospitalization was 2 days (interquartile range, 0–12). Among the hospitalized patients, 5,407 (11.8%) were diagnosed with complicated HZ.

**Table 1:** Demographic and clinical characteristics of individuals with herpes zoster and postherpetic neuralgia

	Herpes zoster (*n* = 692,502)	Postherpetic neuralgia (*n* = 96,450)
Age, median (IQR), years	74.0 (66.0–81.0)	76.0 (70.0–82.0)
Female, n (%)	430,528 (62.2)	58,418 (60.6)
Comorbidities, n (%)		
Asthma	69,894 (10.1)	11,056 (11.5)
Cardiovascular disease	164,132 (23.7)	26,994 (28.0)
Chronic kidney disease	29,008 (4.2)	4,783 (5.0)
Cirrhosis/hepatitis	103,697 (15.0)	16,678 (17.3)
Chronic obstructive pulmonary disease	56,847 (8.2)	10,097 (10.5)
Depression	44,286 (6.4)	5,979 (6.2)
Diabetes mellitus	189,294 (27.3)	30,811 (31.9)
Hematologic malignancies	14,287 (2.1)	2,452 (2.5)
Hematopoietic stem cell transplantation	513 (0.1)	105 (0.1)
HIV infection	266 (0)	26 (0)
Inflammatory bowel disease	2,312 (0.3)	345 (0.4)
Musculoskeletal disorders	232,136 (33.5)	38,595 (40.0)
Neurological disorders	56,646 (8.2)	8,630 (8.9)
Other autoimmune diseases	25,827 (3.7)	4,681 (4.9)
Primary immunodeficiency	12,791 (1.8)	2,421 (2.5)
Psoriasis	5,941 (0.9)	1,041 (1.1)
Rheumatoid arthritis	635 (0.1)	123 (0.1)
Systemic lupus erythematosus	2,641 (0.4)	536 (0.6)
Solid organ transplantation	516 (0.1)	98 (0.1)
Solid tumors	72,213 (10.4)	12,436 (12.9)
Medications, n (%)		
Biologic agents	6,860 (1.0)	1,117 (1.2)
Corticosteroids	83,229 (12.0)	14,574 (15.1)
Immunomodulatory agents	21,914 (3.2)	3,888 (4.0)
Antiviral agents, n (%)		
Acyclovir (intravenous)	25,934 (3.7)	–
Acyclovir (oral)	26,503 (3.8)	–
Amenamevir	214,486 (31.0)	–
Famciclovir	113,720 (16.4)	–
Valacyclovir	309,362 (44.7)	–
Vidarabine	2,497 (0.4)	–

Abbreviation: HIV, human immunodeficiency virus; IQR, interquartile range

Table 2 shows the nine-year trends in the standardized incidence rates of HZ and PHN. Between FY 2014 and FY 2022, the standardized incidence rate of HZ increased from 9.32 per 1,000 person-years in FY 2014 to 10.23 per 1,000 person-years in FY 2022, with an estimated annual percent change of 1.16% (95% CI, 0.52–2.00). The standardized incidence rate of PHN also increased, from 1.01 per 1,000 person-years in FY 2014 to 1.00 per 1,000 person-years in FY 2022, peaking at 1.06 in FY 2021, with an annual percentage change of 0.99% (95% CI, 0.09–3.04). In a sensitivity analysis, when HZ was defined based solely on diagnostic codes, the standardized incidence rate was 10.72 per 1,000 person-years in FY 2014 and 11.31 per 1,000 person-years in FY 2022, corresponding to an annual percent change of 0.71% (95% CI, 0.05–1.59), consistent with the findings of the main analysis.

**Table 2:** Trends in the incidence of herpes zoster and postherpetic neuralgia, fiscal years 2014–2022

	Incidence rate (per 1,000 person-years)									Annual percent change (95% CI)
	2014	2015	2016	2017	2018	2019	2020	2021	2022	
Herpes zoster	9.32	9.37	9.01	9.17	9.47	9.74	9.55	9.80	10.23	1.16% (0.52%–2.00%)
PHN	1.01	0.97	0.91	0.94	1.00	1.04	1.04	1.06	1.00	0.99% (0.09%–3.04%)

Abbreviations: CI, confidence interval; PHN, postherpetic neuralgia

**Fig. 1:**
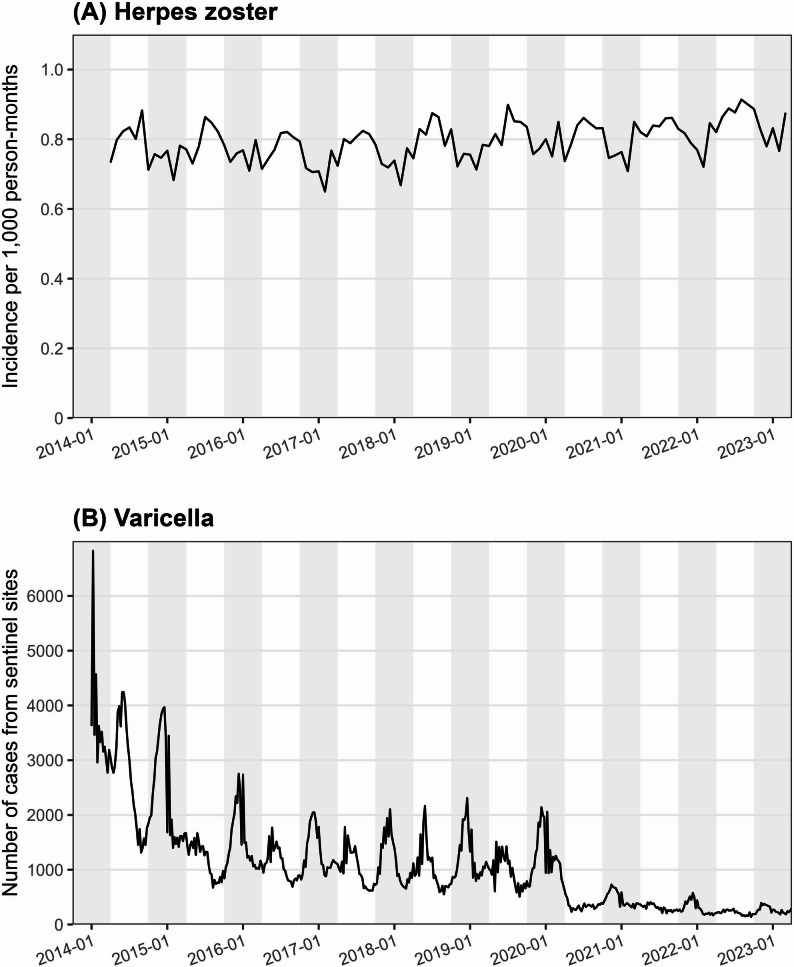
Trends in (**A**) herpes zoster incidence per 1,000 person-months and (**B**) numbers of varicella cases reported at sentinel sites, January 2014–March 2023. Gray-shaded areas indicate winter months (October–March). Herpes zoster showed higher incidence during summer (non-shaded areas), whereas varicella peaked predominantly in winter (gray-shaded areas). Reported varicella cases declined sharply, while herpes zoster incidence showed only a modest increase over the same period

The incidence of HZ was higher in summer, whereas the monthly incidence of varicella declined markedly from January 2014 to March 2023 but remained more common in winter throughout the study period (Fig. 1). The incidence of varicella decreased substantially following the introduction of routine childhood varicella vaccination in Japan in October 2014, with an additional decline observed in 2020 during the coronavirus disease 2019 (COVID-19) pandemic.

**Fig. 2:**
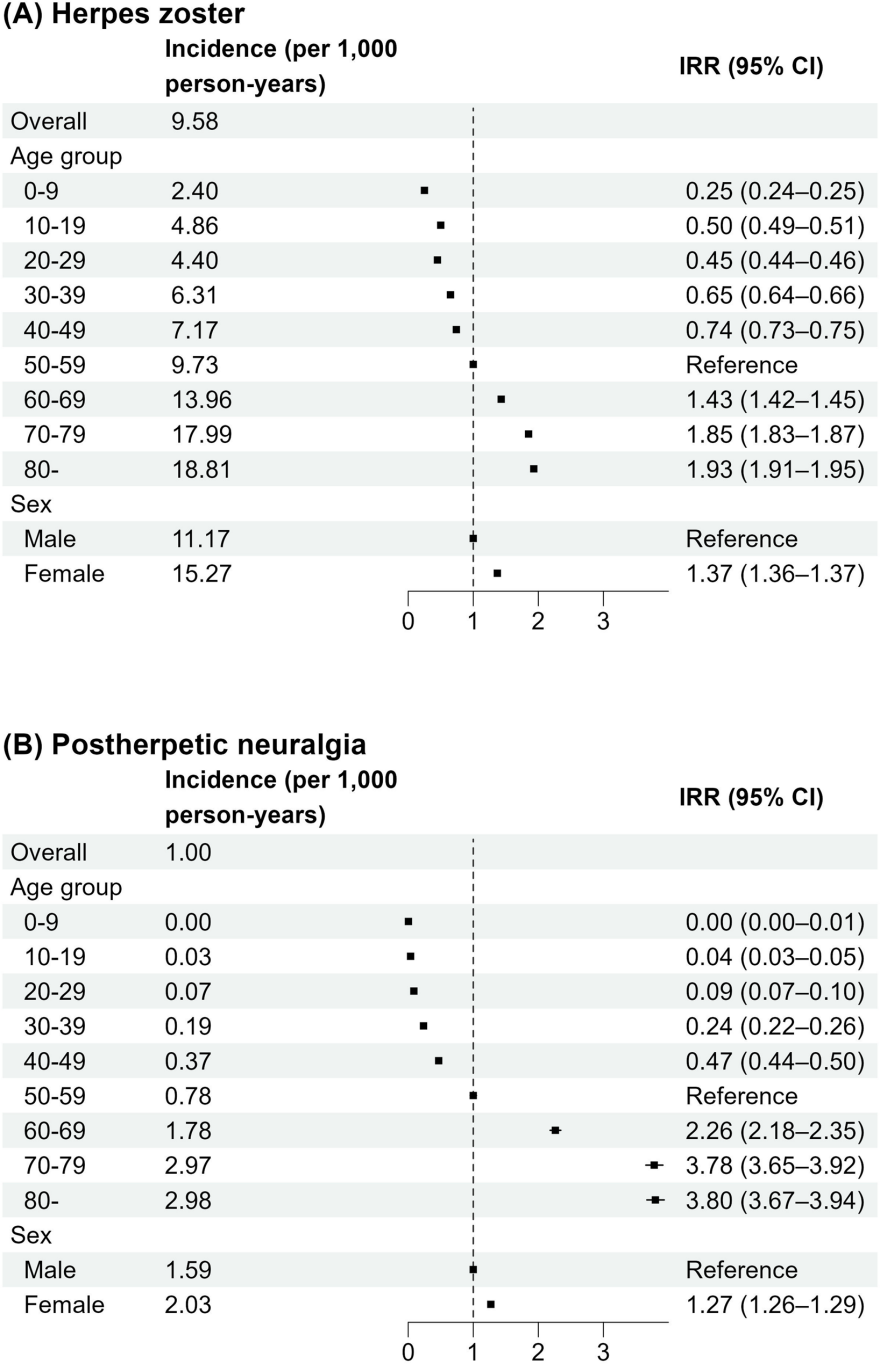
Incidence rates (per 1,000 person-years) and incidence rate ratios of herpes zoster and postherpetic neuralgia. Overall incidence rates were standardized to the 2015 Japanese population by age and sex. . Abbreviations: IRR, incidence rate ratio; CI, confidence interval

Figure 2 shows the incidence rates of HZ and PHN stratified by age and sex. The overall standardized incidence rate of HZ was 9.58 per 1,000 person-years and was higher in females (IRR, 1.37; 95% CI, 1.36–1.37). The incidence increased steadily with age: 2.40 in the 0–9 age group, 4.86 in the 10–19 age group, 4.40 in the 20–29 age group, 6.31 in the 30–39 age group, 7.17 in the 40–49 age group, 9.73 in the 50–59 age group, 13.96 in the 60–69 age group, 17.99 in the 70–79 age group, and 18.81 in those ≥ 80 years. Compared with individuals aged 50–59 years, the IRRs were 1.43 in 60–69 years, 1.85 in 70–79 years, and 1.93 in those aged ≥ 80 years. The overall standardized incidence rate of PHN was 1.00 per 1,000 person-years, and rates were also higher in females (IRR, 1.27; 95% CI, 1.26–1.29). The incidence increased with age: 0.00 in the 0–9 age group, 0.03 in 10–19, 0.07 in 20–29, 0.19 in 30–39, 0.37 in 40–49, 0.78 in 50–59, 1.78 in 60–69, 2.97 in 70–79, and 2.98 in those aged ≥ 80 years. The effect of age was more pronounced for PHN than for HZ; compared with individuals aged 50–59 years, the IRRs were 2.26 in 60–69, 3.78 in 70–79, and 3.80 in those aged ≥ 80 years.

**Fig. 3:**
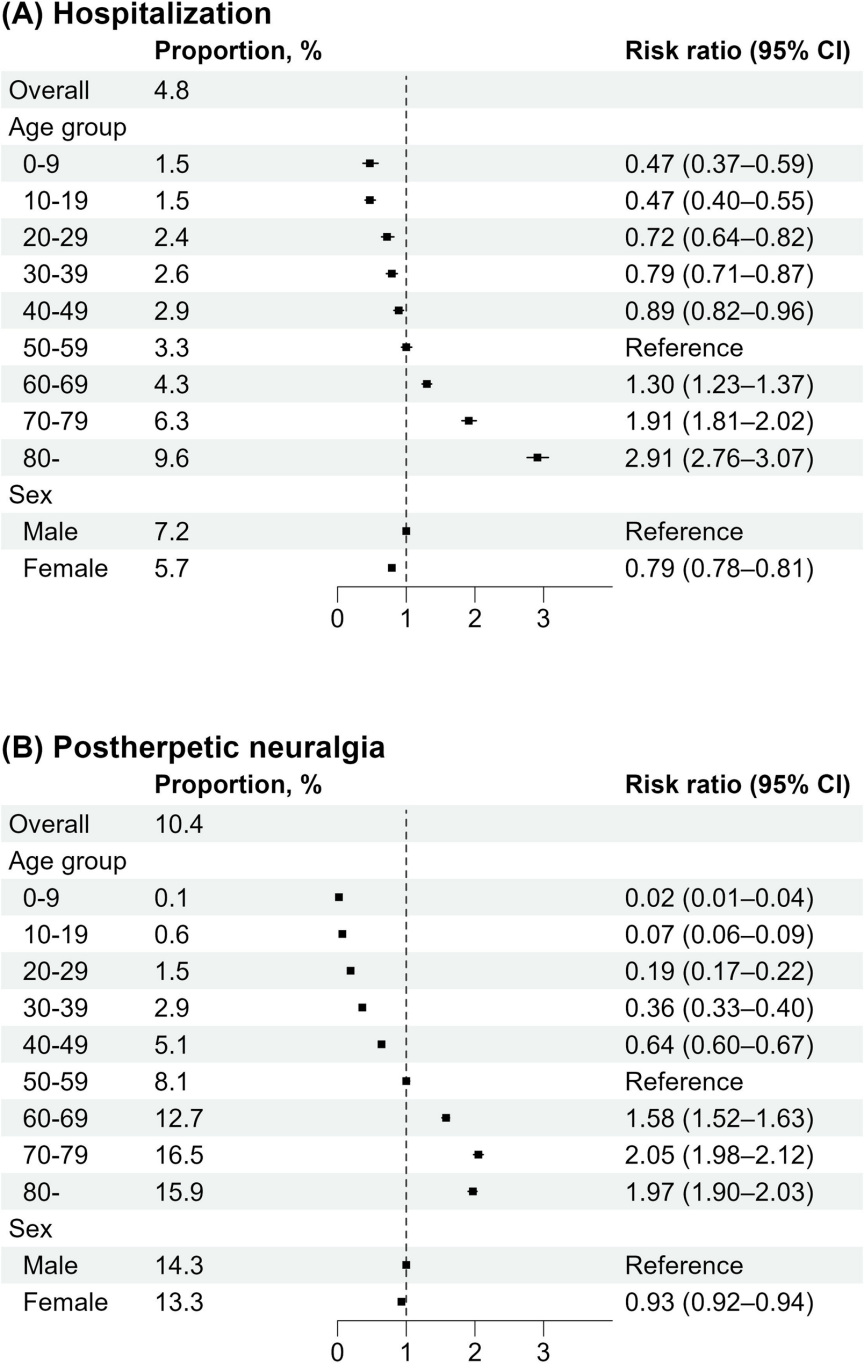
Risks (proportions) and risk ratios for (**A**) hospitalization and (**B**) postherpetic neuralgia after herpes zoster. Overall risks were standardized to the 2015 Japanese population by age and sex. Abbreviation: CI, confidence interval

The risk ratios of complications following HZ, namely hospitalization and PHN, are shown in Fig. 3. The overall standardized risk, expressed as proportions, was 4.8% for hospitalizations and 10.4% for PHN. With individuals aged 50–59 years as the reference group, the risk ratios for hospitalization were 0.47 (95% CI, 0.37–0.59) in the 0–9 age group, 1.30 (95% CI, 1.23–1.37) in 60–69, and 2.91 (95% CI, 2.76–3.07) in those aged ≥ 80 years. For PHN, the corresponding risk ratios were 0.02 (95% CI, 0.01–0.04), 1.58 (95% CI, 1.52–1.63), and 1.97 (95% CI, 1.90–2.03), respectively. In contrast to the incidence rates, the risks of hospitalization and PHN were significantly lower in females than in males, with risk ratios of 0.79 (95% CI, 0.78–0.81) and 0.93 (95% CI, 0.92–-0.94), respectively.

Trends in antiviral prescriptions for HZ are listed in Table 3. In FY 2014, valacyclovir was the most frequently prescribed antiviral agent (50.1%), followed by famciclovir (34.3%). By FY 2022, amenamevir, which was approved in Japan in 2017, had become the most frequently prescribed agent (44.6%), followed by valacyclovir (38.7%) and famciclovir (10.9%).

**Table 3:** Trends in antiviral use for herpes zoster, fiscal years 2014–2022 (% of total prescriptions)

	2014	2015	2016	2017	2018	2019	2020	2021	2022
Antivirals, %									
Acyclovir (IV)	4.5	4.1	5.3	4.4	4.3	3.9	3.5	3.1	2.7
Acyclovir (oral)	9.9	6.8	5.7	4.8	4.5	4.2	3.7	3.6	3.0
Amenamevir	0.0	0.0	0.0	10.4	28.0	32.5	36.0	41.0	44.6
Famciclovir	34.3	32.5	31.3	25.3	16.0	14.4	13.7	11.7	10.9
Valacyclovir	50.1	56.0	57.1	54.5	46.8	44.6	42.8	40.4	38.7
Vidarabine	1.3	0.6	0.6	0.5	0.4	0.3	0.3	0.3	0.2

Abbreviation: IV; intravenous

## Discussion

This study demonstrated that the incidences of HZ and PHN increased with age and were higher in females, with the effect of age being more pronounced in PHN than in HZ. These findings highlight the substantial burden of HZ and PHN in older populations and underscore the importance of effective preventive strategies. Previous studies in Japan reported lower HZ incidence rates than those observed in the present study. A prospective investigation conducted between 2009 and 2015 through a network of dermatology clinics in Miyazaki Prefecture, one of the most important epidemiological studies in Japan, reported an incidence rate of 4.79 per 1,000 person-years [10]. Another analysis based on claims data, primarily from employees’ social health insurance—which underrepresents older individuals—showed an incidence rate of 4.92 per 1,000 person-years between 2005 and 2014 among Japanese adults aged 18 years or older [11]. Because none of these studies comprehensively captured HZ cases, the incidence may have been underestimated. In Japan, where population aging is pronounced and the uptake of HZ vaccines has been delayed, the recent increase in HZ incidence is concerning.

Several epidemiological studies of HZ have been conducted in other East Asian countries. A Taiwanese study using the National Health Insurance register from 2000 to 2006 reported an HZ incidence rate of 4.89 per 1,000 person-years [12]. In South Korea, a population-based analysis of the National Health Insurance Service database showed that adjusted HZ incidence rates increased significantly from 4.23 to 9.22 per 1,000 person-years between 2006 and 2015 [22]. A recent meta-analysis of HZ incidence among Chinese populations across all ages demonstrated a pooled incidence of 4.28 per 1,000 person-years based on six studies conducted between 2012 and 2019 [23]. Additionally, the increasing trend in HZ incidence observed in our study is consistent with a systematic review of international studies from 1945 to 2012, which demonstrated increasing HZ incidence across all age groups both before and after the implementation of varicella vaccination programs [24]. HZ represents a significant global health burden that is expected to rise with the aging population [24], underscoring the importance of preventive measures, including widespread vaccination.

Throughout the study period, the incidence of HZ was consistently higher in summer than in winter, in contrast to varicella, which showed a higher incidence in winter. Similar seasonal patterns were reported in previous studies conducted in Japan and Thailand [25, 26]. The latter study identified a lag of approximately 3 months between the peak of varicella incidence and that of HZ reactivation and demonstrated that HZ incidence was strongly correlated with ambient ultraviolet levels rather than with varicella incidence [26]. In contrast to the sharp decline in varicella incidence during the study period, HZ incidence did not show marked changes. These observations do not support the hypothesis that exposure to varicella boosts VZV-specific cell-mediated immunity, thereby decreasing the risk of HZ [27].

Our study revealed that age was a significant risk factor for HZ and its complications, including hospitalization and PHN. This finding aligns with previous studies that reported higher incidence rates of HZ, hospitalization, and PHN among individuals aged ≥ 50 years [1, 9, 10, 28]. Importantly, our results showed that age was more strongly associated with PHN development than with HZ occurrence. This pronounced effect of aging on PHN is attributable not only to the increased incidence of HZ with advancing age, but also to the higher likelihood of developing PHN following HZ. Accordingly, vaccination against HZ, which reduces both HZ incidence and the risk of PHN, is expected to provide substantial benefits, particularly for individuals aged ≥ 50 years. Our comprehensive epidemiological analysis across all age groups encompassing both HZ and PHN provides valuable evidence for policy decisions regarding the optimal target age for public immunization programs.

The higher incidence rates of HZ and PHN in females were consistent with previous studies [9, 25, 29]. Although the mechanism underlying this sex difference remains unclear, one possible explanation is that females are more likely than males to seek medical care [30]. Fleming et al. reported that while HZ was more common in females, no sex differences were observed for varicella, leading to the hypothesis of potential sex-related differences in immune responses to viral reactivation [31]. In contrast, our results showed a higher risk of PHN (i.e., the probability of developing PHN among individuals with HZ) in males. Similarly, a population-based study in Taiwan reported a slightly higher probability of PHN in males than in females (8.82% vs. 8.37%), consistent with our findings [12].

In database-based studies, PHN is commonly defined as occurring within 30–180 days or 90–180 days after the initial HZ diagnosis; however, both approaches have inherent limitations, and no standardized definition has been established [32]. First, the difficulty in defining PHN in claims-based studies stems from the inability to directly measure pain persistence, necessitating reliance on diagnosis codes and treatment patterns that may be applied before the conventional 90-day threshold is reached [33]. Second, prospective clinical studies can identify even mild pain through active patient assessment, whereas database-based studies are limited to capturing pain that results in healthcare utilization. Third, because NSAIDs and acetaminophen are frequently prescribed for various types of pain other than PHN, they were not included in our definition of PHN, consistent with previous studies [15, 16]. Consequently, patients treated solely with these medications without a specific PHN diagnostic code may not have been identified.

Since 2021, amenamevir has been the most frequently prescribed antiviral agent for HZ treatment. Amenamevir is a newly developed helicase–primase inhibitor approved for the treatment of HZ and recurrent herpes simplex [34]. This once-daily oral agent can be administered without dose adjustments in patients with impaired renal function [35]. Although amenamevir is convenient for outpatient management, the available evidence remains limited to small-scale studies evaluating its effects on acute-phase pain and skin lesions [35, 36], and no studies have assessed its effectiveness in real-world settings. Further research is required to establish its clinical utility.

This study had several limitations. First, individuals with HZ or PHN were identified using diagnostic codes that had not been formally validated. Nonetheless, a previous study demonstrated high concordance between the clinical diagnosis of HZ and polymerase chain reaction (PCR) testing [10]. Second, the database did not include information on HZ vaccination. However, because public subsidization and vaccine uptake were both limited before April 2023 (i.e., during the study period), the potential impact of vaccination on our findings is expected to be minimal. Third, as mentioned above, NSAIDs and acetaminophen were excluded from the definition of PHN-related medications because of their frequent use for a wide range of conditions unrelated to PHN.

## Conclusions

In conclusion, this large-scale epidemiological study demonstrated an increasing trend in the overall incidence of HZ and PHN. Although both HZ and PHN increased with age, the effect of age was more pronounced for PHN than for HZ. The risks of hospitalization and PHN following HZ were substantial, particularly in older adults. HZ incidence peaked in summer, with no clear relationship to varicella incidence. Since 2018, amenamevir and valaciclovir have become the most frequently used agents for the treatment of HZ.

## Supplementary Information

Below is the link to the electronic supplementary material.

Supplementary Material 1

## Data Availability

The raw data used in this study were obtained from DeSC Healthcare and are not publicly available due to ethical restrictions. Analytic datasets are available from the corresponding author upon reasonable request via email.

## Abbreviations

ATC: Anatomical Therapeutic Chemical
CI: Confidence interval
COVID-19: Coronavirus disease 2019
FY: Fiscal year
IRR: Incidence rate ratio
HIV: Human immunodeficiency virus
HZ: Herpes zoster
ICD-10: International Classification of Diseases and Related Health Problems,10th Revision
NSAIDs: Nonsteroidal anti-inflammatory drugs
PCR: Polymerase chain reaction
PHN: Postherpetic neuralgia
VZV: Varicella-zoster virus

## References

[1] Schmader K. Herpes Zoster in older adults. Clin Infect Dis. 2001;32:1481–6. 11317250 10.1086/320169

[2] Johnson RW, Rice AS. Clinical practice. Postherpetic neuralgia. N Engl J Med. 2014;371:1526–33. 25317872 10.1056/NEJMcp1403062

[3] Shin E, Chi SA, Chung TY, Kim HJ, Kim K, Lim DH. The associations of herpes simplex virus and varicella Zoster virus infection with dementia: a nationwide retrospective cohort study. Alzheimers Res Ther. 2024;16:57. 38475873 10.1186/s13195-024-01418-7 PMC10935826

[4] Curhan SG, Kawai K, Yawn B, Rexrode KM, Rimm EB, Curhan GC. Herpes Zoster and Long-Term risk of cardiovascular disease. J Am Heart Assoc. 2022;11:e027451. 36382961 10.1161/JAHA.122.027451 PMC9851464

[5] Thomas SL, Hall AJ. What does epidemiology tell Us about risk factors for herpes zoster? Lancet Infect Dis. 2004;4:26–33. 14720565 10.1016/s1473-3099(03)00857-0

[6] Harbecke R, Cohen JI, Oxman MN. Herpes Zoster vaccines. J Infect Dis. 2021;224(Suppl 4):S429–42. 34590136 10.1093/infdis/jiab387 PMC8482024

[7] Zerbo O, Bartlett J, Fireman B, Lewis N, Goddard K, Dooling K, et al. Effectiveness of Recombinant Zoster vaccine against herpes Zoster in a Real-World setting. Ann Intern Med. 2024;177:189–95. 38190712 10.7326/M23-2023 PMC11001419

[8] Shono A, Hoshi SL, Kondo M. Subsidy programs for herpes Zoster vaccination in Japanese municipalities. Biol Pharm Bull. 2025;48:1185–90. 40769878 10.1248/bpb.b25-00126

[9] Takao Y, Miyazaki Y, Okeda M, Onishi F, Yano S, Gomi Y, et al. Incidences of herpes Zoster and postherpetic neuralgia in Japanese adults aged 50 years and older from a Community-based prospective cohort study: the SHEZ study. J Epidemiol. 2015;25:617–25. 26399445 10.2188/jea.JE20140210 PMC4626391

[10] Shiraki K, Toyama N, Daikoku T, Yajima M. Miyazaki dermatologist Society. herpes Zoster and recurrent herpes Zoster. Open Forum Infect Dis. 2017;4:ofx007. 28480280 10.1093/ofid/ofx007 PMC5414100

[11] Imafuku S, Matsuki T, Mizukami A, Goto Y, de Souza S, Jegou C, et al. Burden of herpes Zoster in the Japanese population with Immunocompromised/Chronic disease conditions: results from a cohort study claims database from 2005–2014. Dermatol Ther (Heidelb). 2019;9:117–33. 30456446 10.1007/s13555-018-0268-8 PMC6380970

[12] Jih JS, Chen YJ, Lin MW, Chen YC, Chen TJ, Huang YL, et al. Epidemiological features and costs of herpes Zoster in taiwan: a National study 2000 to 2006. Acta Derm Venereol. 2009;89:612–6. 19997693 10.2340/00015555-0729

[13] Yasunaga H. DeSC database. Ann Clin Epidemiol. 2025;7:46–9. 40226165 10.37737/ace.25006 PMC11982632

[14] Munoz-Quiles C, Lopez-Lacort M, Orrico-Sanchez A, Diez-Domingo J. Impact of postherpetic neuralgia: A six year population-based analysis on people aged 50 years or older. J Infect. 2018;77:131–6. 29742472 10.1016/j.jinf.2018.04.004

[15] Gialloreti LE, Merito M, Pezzotti P, Naldi L, Gatti A, Beillat M, et al. Epidemiology and economic burden of herpes Zoster and post-herpetic neuralgia in italy: a retrospective, population-based study. BMC Infect Dis. 2010;10:230. 20682044 10.1186/1471-2334-10-230 PMC2921387

[16] Hillebrand K, Bricout H, Schulze-Rath R, Schink T, Garbe E. Incidence of herpes Zoster and its complications in Germany, 2005–2009. J Infect. 2015;70:178–86. 25230396 10.1016/j.jinf.2014.08.018

[17] Japan Society of Pain Clinicians. Practical guidelines for the management of pain. 7th ed. Tokyo (Japan): Bunkodo; 2023. (in Japanese).

[18] Steinmann M, Lampe D, Grosser J, Schmidt J, Hohoff ML, Fischer A, et al. Risk factors for herpes Zoster infections: a systematic review and meta-analysis unveiling common trends and heterogeneity patterns. Infection. 2024;52:1009–26. 38236326 10.1007/s15010-023-02156-y PMC11142967

[19] Sodergren E, Mardberg K, Nishimwe M, Bhavsar A, Marijam A, Bergstrom T, et al. Incidence and burden of herpes Zoster in sweden: A regional Population-Based register study. Infect Dis Ther. 2024;13:121–40. 38193987 10.1007/s40121-023-00902-1 PMC10828402

[20] Sun Y, Miller DC, Akpandak I, Chen EM, Arnold BF, Acharya NR. Association between immunosuppressive drugs and coronavirus disease 2019 outcomes in patients with noninfectious uveitis in a large US claims database. Ophthalmology. 2022;129:1096–106. 35588945 10.1016/j.ophtha.2022.05.008 PMC9110065

[21] Japan Institute for Health Security. Infectious Diseases Weekly Report (IDWR). https://www.niid.go.jp/niid/ja/idwr.html. Accessed 13 Sept 2025.

[22] Choi JK, Park SH, Park S, Cho SY, Lee HJ, Kim SH, et al. The changing epidemiology of herpes Zoster over a decade in South Korea, 2006–2015. Vaccine. 2019;37:5153–60. 31377077 10.1016/j.vaccine.2019.07.086

[23] Zhang Z, Liu X, Suo L, Zhao D, Pan J, Lu L. The incidence of herpes Zoster in china: A meta-analysis and evidence quality assessment. Hum Vaccin Immunother. 2023;19:2228169. 37424092 10.1080/21645515.2023.2228169 PMC10339760

[24] Kawai K, Gebremeskel BG, Acosta CJ. Systematic review of incidence and complications of herpes zoster: towards a global perspective. BMJ Open. 2014;4:e004833. 24916088 10.1136/bmjopen-2014-004833 PMC4067812

[25] Toyama N, Shiraki K, Society of the Miyazaki Prefecture Dermatologists. Epidemiology of herpes Zoster and its relationship to varicella in japan: A 10-year survey of 48,388 herpes Zoster cases in Miyazaki Prefecture. J Med Virol. 2009;81:2053–8. 19856466 10.1002/jmv.21599

[26] Bakker KM, Eisenberg MC, Woods R, Martinez ME. Exploring the seasonal drivers of varicella Zoster virus transmission and reactivation. Am J Epidemiol. 2021;190:1814–20. 33733653 10.1093/aje/kwab073 PMC8579026

[27] Hope-Simpson RE. The nature of herpes zoster: A Long-term study and a new hypothesis. Proc R Soc Med. 1965;58:9–20. 14267505 10.1177/003591576505800106 PMC1898279

[28] Kuniyoshi Y, Tokutake H, Takahashi N, Kamura A, Yasuda S, Tashiro M. Routine varicella vaccination program and hospitalization for herpes Zoster in Japan. Hum Vaccin Immunother. 2021;17:4171–6. 34613868 10.1080/21645515.2021.1971014 PMC8828115

[29] Opstelten W, Van Essen GA, Schellevis F, Verheij TJ, Moons KG. Gender as an independent risk factor for herpes zoster: a population-based prospective study. Ann Epidemiol. 2006;16:692–5. 16516488 10.1016/j.annepidem.2005.12.002

[30] Rowlands S, Moser K. Consultation rates from the general practice research database. Br J Gen Pract. 2002;52:658–60. 12171226 PMC1314386

[31] Fleming DM, Cross KW, Cobb WA, Chapman RS. Gender difference in the incidence of shingles. Epidemiol Infect. 2004;132:1–5. 14979582 10.1017/s0950268803001523 PMC2870070

[32] Yawn BP. Post-shingles neuralgia by any definition is painful, but is it PHN? Mayo Clin Proc. 2011;86:1141–2. 22134931 10.4065/mcp.2011.0724 PMC3228611

[33] Klompas M, Kulldorff M, Vilk Y, Bialek SR, Harpaz R. Herpes Zoster and postherpetic neuralgia surveillance using structured electronic data. Mayo Clin Proc. 2011;86:1146–53. 21997577 10.4065/mcp.2011.0305 PMC3228613

[34] Shiraki K, Yasumoto S, Toyama N, Fukuda H. Amenamevir, a Helicase-Primase Inhibitor, for the optimal treatment of herpes Zoster. Viruses. 2021;13:1547. 34452412 10.3390/v13081547 PMC8402822

[35] Imafuku S, Takeuchi S, Urabe K, Arakawa M, Sasaki R, Oka D, et al. An exploratory study of the efficacy and safety of amenamevir for the treatment of herpes Zoster in patients receiving immunosuppressive drugs. J Dermatol. 2024;51:1279–89. 39046277 10.1111/1346-8138.17364 PMC11483900

[36] Kageshima Y, Inada E, Yamaguchi K, Hayashida M. A comparison between effects of amenamevir and Famciclovir on intensities of acute pain and the incidence of postherpetic neuralgia in adult patients with herpes Zoster. Juntendo Iji Zasshi. 2022;68:120–30. 38912280 10.14789/jmj.JMJ21-0036-OA PMC11189787

