# Supplementary material for: Epidemiology of herpes zoster and postherpetic neuralgia in Japan: analysis of a large-scale claims database
Source: BMC Infect Dis. 2026 Jan 10;26:287. doi: 10.1186/s12879-026-12534-0 (PMC12882626; doi:10.1186/s12879-026-12534-0)
Supplement: Supplementary file 1 — Supplementary Material 1 [file 12879_2026_12534_MOESM1_ESM.docx]

Supplementary Table 1. Classifications and ICD-10 codes of comorbidities.

| Disease category | ICD-10 codes |
| --- | --- |
| Asthma | J45, J46 |
| Cardiovascular disease | I11, I130, I132, I139, I20–25, I44–49 |
| Chronic kidney disease | N18 |
| Cirrhosis/hepatitis | B15–19, K73–76 |
| Chronic obstructive pulmonary disease | J41–44 |
| Depression | F31–34, F38, F39 |
| Diabetes mellitus | E10, E11, E14 |
| Hematologic malignancies | C81–96 |
| Hematopoietic stem cell transplantation | Z948 (including only hematopoietic stem cell transplantation-related status) |
| HIV infection | B21–24 |
| Inflammatory bowel disease | K50, K51 |
| Musculoskeletal disorders | M15–19, M80, M81 |
| Neurological disorders | G35, I61–64 |
| Other autoimmune diseases | E063, I01, J990, J991, L10–12, L405, L920, M061, M30, M31, M33, M34, M350–353, M359, M36, M941 |
| Primary immunodeficiency | D60, D61, D70–72, D761, D80–84, D89, G113 |
| Psoriasis | L40 (excluding L405), M070–073, M090 |
| Rheumatoid arthritis | M059, M060, M068 |
| Systemic lupus erythematosus | M32 |
| Solid organ transplantation | T861–864, T868 (including only lung transplantation-related status), Z940–944, Z948 (including only small bowel and pancreas transplantation-related status), Z949 |
| Solid tumors | C00–26, C30–41, C43–47, C49–58, C60–68, C73–80 (excluding C762) |

Abbreviation: HIV, human immunodeficiency virus.

Supplementary Table 2. Classification of immunomodulatory agents and biologic agents with codes of ATC Classification System.

| Category | Drugs (codes of ATC Classification System) |
| --- | --- |
| Immunomodulatory agents |  |
| Disease-modifying antirheumatic drugs | Azathioprine (L04AX01), cyclosporine (L04AD01), leflunomide (L04AK01), methotrexate (L04AX03),  mycophenolic acid (L04AA06), tacrolimus (L04AD02) |
| Other immunomodulatory agents | Apremilast (L04AA32), baricitinib (L04AF02), cladribine (L04AA40), cyclophosphamide (L01AA01), dimethyl fumarate (L04AX07), everolimus (L04AH02), filgotinib (L04AF04), fingolimod (L04AE01), glatiramer acetate (L03AX13), immunoglobulin (J06BA), mercaptopurine (L01BB02), ruxolitinib (L01EJ01), siponimod (L04AE03), sirolimus (L04AH01), sulfasalazine (A07EC01), tofacitinib (L04AF01), upadacitinib (L04AF03) |
| Biologic agents |  |
| Interleukin-6 inhibitors | Sarilumab (L04AC14), satralizumab (L04AC19), tocilizumab (L04AC07) |
| Tumor necrosis factor-alpha inhibitors | Adalimumab (L04AB04), certolizumab pegol (L04AB05), etanercept (L04AB01), golimumab (L04AB06), infliximab (L04AB02) |
| Other biologic agents | Abatacept (L04AA24), alemtuzumab (L04AG06), basiliximab (L04AC02), belimumab (L04AG04), brodalumab (L04AC12), canakinumab (L04AC08), dupilumab (D11AH05), eculizumab (L04AJ01), guselkumab (L04AC16), inebilizumab (L04AG10), interferon beta-1a (L03AB07), interferon beta-1b (L03AB08), ixekizumab (L04AC11), natalizumab (L04AG03), ofatumumab (L01FA02), omalizumab (R03DX05), ravulizumab (L04AJ02), risankizumab (L04AC18), rituximab (L01FA01), secukinumab (L04AC10), tildrakizumab (L04AC17), ustekinumab (L04AC05), vedolizumab (L04AG05) |

Abbreviation: ATC, anatomical therapeutic chemical.

Supplementary Table 3. Age-stratified distribution of the study population in 2022 and the general population of Japan in 2015

| Age group (years) | Study population, 2022, n (%) | General population of Japan, 2015, n (%) |
| --- | --- | --- |
| 0–9 | 244,529 (2.7) | 10,325,000 (8.1) |
| 10–19 | 299,710 (3.3) | 11,674,000 (9.2) |
| 20–29 | 460,142 (5.1) | 12,623,000 (9.9) |
| 30–39 | 446,672 (4.9) | 15,813,000 (12.4) |
| 40–49 | 568,342 (6.3) | 18,613,000 (14.6) |
| 50–59 | 632,560 (7.0) | 15,625,000 (12.3) |
| 60–69 | 1,186,528 (13.1) | 18,311,000 (14.4) |
| 70–79 | 2,820,974 (31.1) | 14,141,000 (11.1) |
| ≥ 80 | 2,403,655 (26.5) | 9,969,000 (7.8) |
